# Associations between PM_2.5_ exposure and Alzheimer’s Disease prevalence Among elderly in eastern China

**DOI:** 10.1186/s12940-022-00937-w

**Published:** 2022-11-29

**Authors:** Li Yang, Wenjie Wan, Caiyan Yu, Cheng Xuan, Pinpin Zheng, Jing Yan

**Affiliations:** 1 Zhejiang Hospital, No.12 Ling Yin Road, Hangzhou, 310013 China; 2 Key Laboratory of Public Health Safety, Ministry of Education, Health Communication Institute, Fudan University, 138 Yixueyuan Road, Shanghai, 200032 China; 3 Zhejiang Chinese Medical University, Hangzhou, China; 4 Zhuji Second People’s Hospital, No. 15 Fengbei Road, Fengqiao Town, Zhuji, 311811 China

**Keywords:** Alzheimer’s Disease, PM_2.5_ exposure, Association

## Abstract

**Background:**

Studies showed that PM_2.5_ might be associated with various neurogenic diseases such as Alzheimer’s Disease (AD). However, this topic had been little studied in Zhejiang province of China.

**Methods:**

In 2018, we established a cohort of AD high-risk population with 1,742 elderly aged 60 and above. In 2020, the cohort was followed up, a total of 1,545 people participated the 2 surveys. Data collection included questionnaires and basic physical examinations. The average residential exposure to PM_2.5_ for each participant, that in a 5-years period prior to the first survey, was estimated using a satellite-based spatial statistical model. We determined the association between PM_2.5_ and AD prevalence by cox proportional hazards regression model.

**Results:**

This study showed that an increase in the PM_2.5_ level was an important associated risk factor that contributed to AD. The average PM_2.5_ exposure levels among the study population ranged from 32.69 μg/m^3^ to 39.67 μg/m^3^ from 2013 to 2017, which were much higher than 5 μg/m^3^ that specified in the WHO air quality guidelines. There was an association between PM_2.5_ exposure and AD, and the correlations between PM_2.5_ and Mini-Mental State Examination, Montreal cognitive assessment scale scores were statistically significant. An increase in the PM_2.5_ level by 10 μg/m^3^ elevated the risk of AD among residents by 2%-5% (HR _model 2-model 4_ = 1.02 to 1.05, CI _model 2-model 4_ = 1.01–1.10). The subgroups of male, with old age, with low education levels, used to work as farmers or blue-collar workers before retirement, overweight and obese were associated with a higher effect of PM_2.5_.

**Conclusions:**

Reducing PM_2.5_ exposure might be a good way to prevent AD.

**Supplementary Information:**

The online version contains supplementary material available at 10.1186/s12940-022-00937-w.

## Introduction

With the development of social economy, the health hazards of particulate air pollution have become an important public health concern to governments around the world and the World Health Organization [1].

Air pollution is a kind of complicated mixture, comprising particulate matter (PM), carbon monoxide, nitrogen dioxide, ozone, sulfur dioxide and so on. PM_2.5_, the small PM with diameter less than 2.50 µm, is now regarded as one of the most harmful factors to our health [2].

According to the data analysis of WHO, in 2016 about 4.20 million people suffered from air pollution, leading to shorter lifespan, which was mostly resulted from the PM_2.5_ [2]. With a small particle size, it could penetrate into the lower respiratory tract and the blood circulation via alveolar capillaries of a person, causing health damage to the cardiovascular system, the respiratory system and more other human systems [3]. The Global Burden of Disease study showed that air pollution exposure caused 2.94 million premature deaths in 2017, ranking as the fifth leading risk factor for global mortality [4].

On September 2021, the WHO has published an update of the global Air Quality Guidelines (AQG 2021). In the 15 years that separate the new document from the previous edition (2005), the quality and quantity of studies documenting the negative influence of air pollution on health have considerably increased. For this reason, and after a systematic review of the accumulated evidence, the updated AQG values are lower than those recommended 15 years ago; particularly, the average annual concentrations of PM_2.5_ decrease from 10 to 5 μg/m^3^ [5].

In China, PM_2.5_ exposure claimed a total of 850,000 lives, which accounted for 29% of the total deaths in the world. As the largest developing country in the world, China was faced with severe air pollution problems. The annual average PM_2.5_ in China was far above 5 μg/m^3^ which specified in the WHO air quality guidelines [5].

Alzheimer’s disease (AD) was a chronic central nervous system disorder that developed progressively from insidious onset, characterized by progressive memory impairment and cognitive loss, accompanied by a decreasing ability and behavioral changes. The incidence of this most common type of dementia increased with age. According to the *2020 Alzheimer’s Disease Facts and Figures*, about 50 million people in the world suffered from AD or other types of dementia, compared with 152 million by 2050 [6]. The National Institute on Aging found that AD was the third leading cause of death for elderly, second only to heart disease and cancer in America. In China, more than 15 million people suffered from dementia (including 9.83 million AD patients) [7].

In neurodegenerative diseases, environmental factors played a key role in the development of AD. A recent 2020 Lancet Commission on dementia prevention, intervention, and care identified air pollution as one of 12 modifiable risk factors that could prevent or delay dementia [8, 9]. Estimates suggested about 2.10 million incident dementia cases could be attributable to ambient exposure to PM_2.5_ pollution in 2015 [10]. The relationship between PM_2.5_ and cognitive functions could be based on several proposed biological mechanisms, including cerebrovascular injury, neuroinflammation, and neurodegeneration [11]. Air pollutants could directly elicit inflammatory changes and oxidative stress in brain and increase the risk of cardio metabolic diseases, ultimately increasing the risks of dementia and cognitive decline [12, 13]. It seemed that small particles from burning fossil fuels were a possible important source of protein toxic stress that caused unbalanced protein folding homeostasis [14].

Large-scale of epidemiological investigation have revealed that exposure to air pollution, especially the PM imposed adverse impact on the cognition of human-beings [14–16]. However, there were little studies about air pollution and dementia including of AD in China. In this study, we aimed to explore the relationship between PM_2.5_ exposure and AD among elderly in eastern China.

## Methods

### Population and data collection

**Fig. 1:**
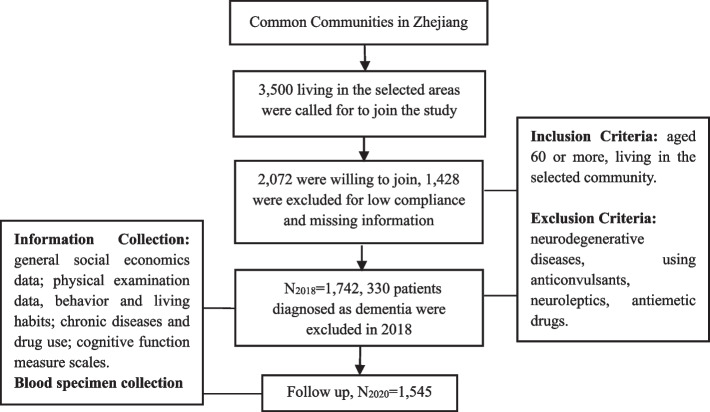
Follow-up flow chart of elderly AD Cohort in Zhejiang Province

We established a cohort of AD high-risk population in Zhejiang Province. Twelve administrative districts of Zhejiang province were divided into 4 groups based on economic levels [17]. From each of these 4 groups, 1 district was systematically selected. Then 1 community was randomly chosen from each district. Subjects met the following criteria were invited to participate: age ≥ 60, living in the selected community for more than 5 years, with normal cognitive function. Exclusion criteria: other types of neurodegenerative diseases such as vascular cognitive impairment, dementia with Lewy bodies, parkinson’s dementia, using anticonvulsants, neuroleptics, antiemetic drugs. Follow-up Flow Chart of the Cohort was showed in Fig. 1.

### Questionnaire and basic physical examination

The survey was conducted by trained general practitioners in community health service centers or participants' residences. Data collection included questionnaires and basic physical examinations. The questionnaires was designed by the National Center for Cardiovascular Disease, which was used in sub-centers throughout the country. There were mainly two parts of the questionnaires included of basic information and preliminary screening questionnaire and follow-up questionnaire, which contained hundreds of questions. We collected basic information of participants including age, education level, profession, and geocoded residential addresses, and so on. Basic physical examination included height, weight, BMI, waist, blood pressure, heart rate, blood tests including high-density lipoprotein (HDL), triglyceride (TG), cholesterol (TC), low density lipoprotein (LDL), glucose (GLU) and urine tests. Scales such as Mini-Mental State Examination (MMSE), Montreal cognitive assessment scale (MoCA) used in this study were the most common rapid screening tools for cognitive dysfunction in the world. The scale were translated and revised into many languages and widely used in clinical practice. Chinese version of them were confirmed having good reliability and validity.

The cognitive status was assessed combining MMSE, MoCA and the Hospital Anxiety and Depression Scale (HAD) [18]. Particularly, MMSE, the most commonly used instrument to screen cognitive impairment, showed education and language/cultural bias and it usually took about 5 min to complete [19]. MoCA was more sensitive in the screening of mild cognitive impairment, especially in cases with impairment of a single cognitive domain, such as amnestic cognitive impairment, and it took about 10 min to complete [20]. Using the combination of MMSE and MoCA had brought a number of benefits: firstly, it improved the accuracy of cognitive impairment screening, and could initially perform simple screening for various cognitive disorders. Secondly, it improved the detection rate of cognitive impairment, increased sensitivity and specificity, and reduced false positive rate and false negative rate. The HAD scale was a primary survey of depression and anxiety [18]. The diagnosis process was conducted by specifically trained psychiatrists based on guidelines [20], combined with the results of the MMSE and MoCA, magnetic resonance imaging (MRI) was used when needed.

Hypertension was defined as a mean systolic pressure (SBP) of at least 140 mm Hg or a mean diastolic pressure (DBP) of at least 90 mm Hg, or use of an antihypertensive drug in the past 2 weeks. Physical examination was conducted in accordance with standard procedures, mainly including height and weight. Body mass index (BMI) was defined as weight (kg) divided by height ^2^ (m^2^). Normal was defined as BMI < 24 kg/m^2^, overweight was defined as BMI ≥ 24 kg/m^2^ and < 28 kg/m^2^, obesity was defined as BMI ≥ 28 kg/m^2^[21].Smoking was defined as continuous or cumulative smoking for 6 months or more, while alcohol consumption was defined as drinking at least 2 times a week.

Quality checks were carried out on the measures. In fact, when measuring height and weight, participants were asked to wear light clothing, no shoes. Blood pressure was measured in a sitting position, resting for at least 5 min before measurement, which was measured twice on the right upper arm using a standard electronic sphygmomanometer (Omron HEM-7430). If the difference between two readings was greater than 10 mm Hg, a third measurement was taken and the average of the last two readings was used. MMSE test including of Chinese version was confirmed that having good reliability and validity [22].

### Air pollution exposure assessment

The geocoded residential addresses of 1,545 participants were linked to average PM_2.5_ concentrations between 2013 and 2017, which were estimated from a satellite based spatial statistical model developed by Ma et al. [23]. Briefly, this model was established using the collection 6 aerosol optical depth (AOD) retrieved by the US National Aeronautics and Space Administration (NASA) Moderate Resolution Imaging Spectroradiometer (MODIS), assimilated meteorology data, land use data (fire spots, urban and forest cover, etc.) and PM_2.5_ concentrations from Chinese ground monitoring network [24]. This model was validated to have little bias in the monthly estimates on PM_2.5_. For a certain grid cell, the model could not predict the PM_2.5_ value if the AOD value was missing. A minimum of 6 data points of AOD in a month was showed to be sufficient to appropriately represent a monthly average [23].

The geocoding and exposure assignment was conducted in ArcMap (Version 10.2) [24]. Specifically, we merged the grid cells of modeled data over the study period with the boundaries of Chinese administrative divisions. Each grid cell had a spatial resolution of 1 km × 1 km, with individuals who resided in the same cell sharing the same exposure levels. The modeled exposures were recorded as monthly averages, and we calculated the average concentrations one to five years before the first physical examination (2013 to 2017). And the average concentrations was used as indicators of the historical (long-term) exposure. For sensitivity analysis, we obtained another source of modeled PM_2.5_ concentrations from the Global Burden of Disease database, which generated yearly average estimates by combining the satellite-based estimates, chemical transport model simulations and ground measurements [25].

### Ethics

This study was approved by the Scientific and Ethical Committee of Zhejiang Hospital. All participants were informed of the purpose and method of the study and signed the informed consent.

### Statistical analysis

Epidata 3.0 was used for data entry, and SAS 9.4 was used for data management and analysis. Frequency (percentage) description was used for counting data, and mean and standard deviation description was used for measuring data, such as socio-demographic characteristics including of age, BMI, waistline, laboratory test results including of SBP, DBP, HDL, TG, TC, LDL and cognitive function of the subjects. T test and chi-square test were used to compare the statistical differences for measuring data and measuring data, respectively. A common health association analysis method used with follow-up data in prospective cohort studies is cox proportional hazards regression model, the ratio of any two risk functions refers to the relative hazard (HR).

We built a unadjusted model and three adjusted models. Model 1 was an unadjusted model; Model 2 included PM_2.5_, age, gender; Model 3 added smoking and environmental tobacco smoke (ETS) exposure based on Model 2; Model 4 added educational degree, family income, BMI, and occupation before retirement based on Model 3.

To test the possible effect modification, we conducted several stratification analyses by age groups, sex, educational level, marital status, occupation and BMI, using adjusted models including variables above except stratification variables. All analyses were conducted by bilateral significance test, and the significance level of hypothesis test was set to *P* < 0.05.

## Results

### Baseline information

From March to July of 2018, 3,500 subjects living in the selected areas were called for to join our study, and 2,072 wanted to join. 330 patients diagnosed as dementia in 2018 were excluded, 1,742 subjects were included in the cohort. In 2020, subjects in the cohort were followed up, and 1,545 cases finished the 2 interviews in 2018 and 2020. The follow-up rate was 88.69%. There was no statistical difference of major sociodemographic variables between participants and non-participants, including of age, gender, education level (Supplementary Table 1). The baseline information of the cohort was shown in Table 1.

**Table 1:** Baseline Information of the Cohort [n (%)/Mean ± Standard Deviation]

Variable	Classification	Total (*n* = 1545)	Male (*n* = 739)	Female (*n* = 806)	*P* values
Age		68.21 (4.81)	68.62 (4.91)	67.72 (4.60)	< 0.001
Age group	60–64	403 (26.08)	174 (23.55)	229 (28.41)	0.001
	65–69	528 (34.17)	243 (32.88)	285 (35.36)	
	70–74	436 (28.22)	214 (28.96)	222 (27.54)	
	75–85	178 (11.52)	108 (14.61)	70 (8.68)	
Marital status	Living alone	205 (13.65)	63 (8.82)	142 (18.02)	< 0.001
	Cohabitation	1297 (86.35)	651 (91.18)	646 (81.98)	
Annual household income	< 50,000	309 (20.59)	134 (18.77)	175 (22.24)	0.11
	50,000–100,000	882 (58.76)	439 (61.48)	443 (56.29)	
	> 100,000	310 (20.65)	141 (19.75)	169 (21.47)	
Educational degree	Illiteracy	232 (15.02)	61 (8.25)	171 (21.22)	< 0.001
	Primary school	910 (58.90)	433 (58.59)	477 (59.18)	
	Junior high school	350 (22.65)	215 (29.09)	135 (16.75)	
	Senior high school and above	53 (3.40)	30 (4.06)	23 (2.85)	
Occupation before retirement	Farmers	739 (47.83)	343 (46.41)	396 (49.13)	< 0.001
	Blue-collar workers	705 (45.63)	328 (44.38)	377 (46.77)	
	White-collar workers	101 (6.54)	68 (9.20)	33 (4.09)	
BMI, kg/m^2^		24.51 (3.02)	24.36 (2.88)	24.54 (3.18)	0.55
BMI group					0.15
BMI < 24	Normal	689 (45.93)	329 (46.08)	360 (45.80)	
BMI ≥ 24 and < 28	Overweight	167 (11.13)	68 (9.52)	99 (12.60)	
BMI ≥ 28	Obese	644 (42.93)	317 (44.40)	327 (41.60)	
Smokers	No	1189 (76.96)	450 (60.89)	739 (91.69)	< 0.001
	Yes	356 (23.04)	289 (39.11)	67 (8.31)	
ETS	No	995 (64.40)	432 (58.46)	563 (69.85)	< 0.001
	Yes	550 (35.60)	307 (41.54)	243 (30.15)	
Anxiety	No	1360 (96.25)	634 (96.65)	726 (95.90)	0.49
	Yes	53 (3.75)	22 (3.35)	31 (4.10)	
Depression	No	1255 (81.34)	620 (83.90)	635 (78.98)	0.02
	Yes	288 (18.66)	119 (16.10)	169 (21.02)	
Waistline	Normal	488 (32.53)	298 (41.74)	190 (24.17)	< 0.001
	Abnormal	1012 (67.47)	416 (58.26)	596 (75.83)	
SBP, mmHg		150.71 (20.02)	149.92 (19.33)	151.37 (20.64)	0.15
	< 140	451 (30.00)	227 (31.80)	224 (28.50)	0.23
	140–18	927 (61.80)	435 (60.90)	492 (62.50)	
	≥ 180	123 (8.20)	52 (7.30)	71 (9.0)	
DBP, mmHg		81.32 (10.73)	83.24 (11.08)	79.68 (10.12)	< 0.001
	< 90	1184 (78.88)	519 (72.69)	665 (84.50)	< 0.001
	< 110	307 (20.45)	188 (26.33)	119 (15.12)	
	≥ 110	10 (0.67)	7 (0.98)	3 (0.38)	
HDL, mmol/L	≥ 1.0	75 (5.09)	32 (4.58)	43 (5.54)	0.477
	< 1.0	1399 (94.91)	666 (95.42)	733 (94.46)	
TG, mmol/L	< 1.7	1002 (69.98)	504 (76.13)	498 (64.93)	< 0.001
	≥ 1.7	427 (30.02)	158 (23.87)	269 (35.07)	
TC, mmol/L	< 5.7	1095 (73.10)	576 (80.79)	519 (66.11)	< 0.001
	≥ 5.7	403 (26.90)	137 (19.21)	266 (33.89)	
LDL, mmol/L	< 3.3	1234 (82.27)	628 (88.08)	606 (77.00)	< 0.001
	≥ 3.3	266 (17.73)	85 (11.92)	181 (23.00)	

*Abbreviations*: *BMI* Body Mass Index, *ETS* Environmental Tobacco Smoke, *SBP* Systolic blood pressure, *DBP* Diastolic blood pressure, *HDL* High-density lipoprotein, *TG* triglyceride, *TC* cholesterol, *LDL* Low density lipoprotein

### Analysis of PM_2.5_ exposure

**Fig. 2:**
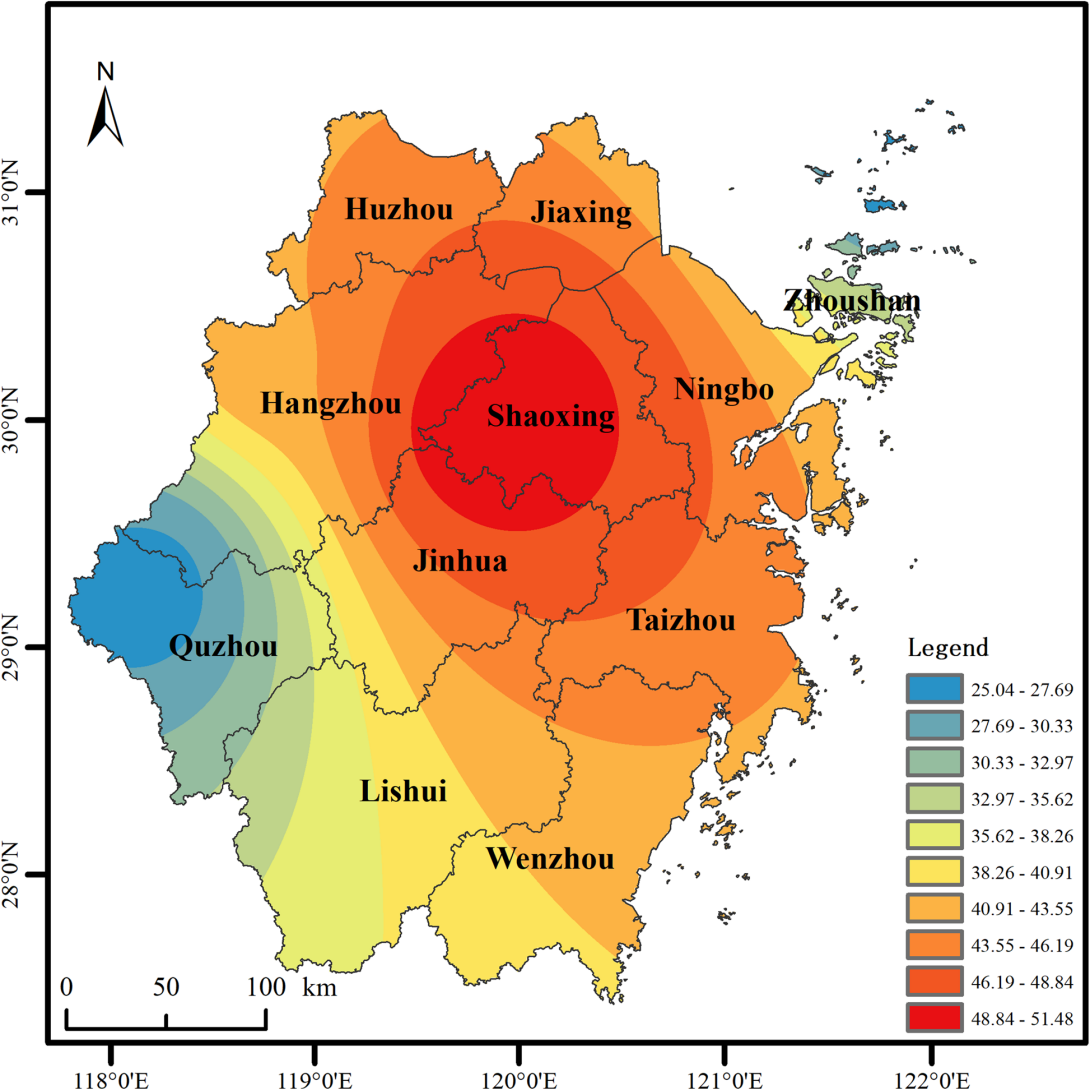
A map of PM_2.5_ exposure relating to the concentration attributed to residents

Table 2 summarized the average air pollution data on the residential addresses of the study population baseline (*n* = 1,545). The average PM_2.5_ exposure level of the study population from 2013 to 2017 ranged from 32.69 μg/m^3^ to 39.67 μg/m^3^, which was far above 5 μg/m^3^ specified in the WHO air quality guidelines [5]. Figure 2 showed an exposure PM_2.5_ map relating to the concentration attributed to residents using the 10 × 10 km grid.

**Table 2:** Summary of Air Pollution Date (2013–2017)

Exposure Year	Percentiles						
	**Mean ± Standard Deviation**	**Min**	**Max**	**25th**	**50th**	**75th**	**IQR**
2017	32.69 ± 1.76	24.98	38.97	32.06	33.25	33.71	1.65
2016	34.72 ± 1.80	27.20	41.68	34.14	35.28	35.84	1.72
2015	35.89 ± 2.09	27.41	44.24	35.17	36.25	37.14	1.97
2014	37.76 ± 2.49	28.24	45.68	36.81	38.00	39.08	2.27
2013	39.67 ± 2.95	29.13	46.42	38.48	39.93	41.34	2.86

*Abbreviations*: *25th* 25th percentile, *50th* 50th percentile, *75th* 75th percentile, *IQR* the inter quartile range

### Analysis of effects on PM_2.5_ exposure

Table 3 described the association between PM_2.5_ pollution and the incidence of AD. It was expressed in the average effect caused by an increase in particle concentration by 10 μg/m^3^ and its 95% confidence interval. The integration analysis showed that the increase in PM_2.5_ level by 10 μg/m^3^ could elevate the risk of AD by 2%-5% among the residents in adjusted models (HR = 1.02 to 1.05).

**Table 3:** Association between PM_2.5_ exposure (per 10 μg/m^3^ increment) and incidence of AD

Model	HR	95% CI	*P* values
Model 1	0.85	0.76–1.05	0.06
Model 2	1.02	1.01–1.09	0.04
Model 3	1.05	1.02–1.11	0.03
Model 4	1.03	1.01–1.10	0.04

*Abbreviations*: *HR* relative hazard, *95%CI* 95% confidence interval, *AD* Alzheimer's Disease

^a^Model 1 was an unadjusted model; Model 2 included PM_2.5_, age, gender; Model 3 added smoking and ETS exposure based on Model 2; Model 4 added educational degree, family income, BMI, and occupation before retirement based on Model 3

The correlations between PM_2.5_ and MMSE and MoCA scores were statistically significant. In the adjusted models, when PM_2.5_ was increased by 10 μg/m^3^, the MMSE and MoCA scores in the cognitive function screening scale decreased by 0.35–0.38 and 0.14–0.16, respectively, pointing to declining cognitive functions, as showed in Table 4.

**Table 4:** Analysis on the Relationship between Cognitive Function Score and PM_2.5_ Exposure

Model	MMSE	MoCA	*P* values for MMSE	*P* values for MoCA
Model 1	0.35 (0.18–0.51)	0.14 (0.08–0.18)	0.03	0.02
Model 2	0.38 (0.19–0.54)	0.15 (0.10–0.22)	0.04	0.03
Model 3	0.36 (0.26–0.47)	0.16 (0.11–0.21)	0.02	0.01
Model 4	0.37 (0.21–0.45)	0.15 (0.08–0.24)	0.03	0.03

^a^Model 1 was an unadjusted model; Model 2 included PM_2.5_, age, gender; Model 3 added smoking and ETS exposure based on Model 2; Model 4 added educational degree, family income, BMI, and occupation before retirement based on Model 3

### Results of stratification analysis

To test the possible effect modification, we conducted several stratification analyses by age groups, sex, educational level, marital status, occupation and BMI, using adjusted models including variables above except stratification variable itself. Table 5 showed the results of the association between PM_2.5_ Exposure (Per 10 μg/m3) and AD prevalence by subgroups. It showed that subgroups of male, with older age, with lower educational level, those who worked as blue-collar workers or farmers before retirement, with a higher BMI (overweight, obesity) were associated with a higher effect of PM_2.5_.

**Table 5:** Analysis on the association between PM_2.5_ Exposure (Per 10 μg/m^3^) and AD prevalence by subgroups

Variables	Group	HR (95% CI)	*P* values
Gender	Male	1.03 (1.01–1.12)	0.03
	Female	0.94 (0.81–1.08)	0.10
Age group, years	60–64	0.96 (0.72–1.24)	0.13
	65–69	1.04 (0.98–1.12)	0.15
	70–74	1.11 (1.10–1.52)	0.02
	75–85	1.12 (1.11–1.43)	0.02
Education level	Illiteracy	1.13 (1.09–1.20)	0.03
	Primary school	1.01 (0.99–1.15)	0.04
	Junior high school	0.98 (0.77–1.24)	0.23
	Senior high school and above	0.86 (0.26–2.81)	0.31
Marital status	Living alone	1.01 (0.89–1.14)	0.52
	Cohabitation	0.76 (0.58–1.00)	0.78
Occupation	Farmers	1.02 (1.00–1.16)	0.04
	Blue-collar	1.09 (1.01–1.25)	0.02
	White-collar	0.65 (0.43–1.12)	0.42
BMI, kg/m^2^	Normal, BMI < 24	0.89 (0.76–1.03)	0.36
	Overweight, BMI ≥ 24 and < 28	1.04 (1.01–1.27)	0.04
	Obese, BMI ≥ 28	1.03 (1.00–1.12)	0.04

*Abbreviations*: *HR* relative hazard, *95% CI* 95% confidence interval, *AD* Alzheimer's Disease

## Discussion

Some studies showed that, environmental risks played a key role in the progression of dementia, PM_2.5_ was an important factor that contributed to dementia including AD [14, 26–30]. On September 2021, the WHO has published an update of the global AQG 2021. In the 15 years that separate the new document from the previous edition in 2005, the quality and quantity of studies documenting the negative influence of air pollution on health have considerably increased. For this reason, and after a systematic review of the accumulated evidence, the updated AQG values are lower than those recommended 15 years ago, particularly, the average annual concentrations of PM_2.5_ decrease from 10 to 5 μg/m^3^ [5]. This study showed that the average PM_2.5_ exposure levels among the study population ranged from 32.69 μg/m^3^ to 39.67 μg/m^3^ from 2013 to 2017, far above 5 μg/m^3^ specified in the WHO air quality guidelines. There was an association between PM_2.5_ exposure and AD, and the correlations between PM_2.5_ and MMSE and MoCA scores were also statistically significant. In the adjusted models, an increase in the PM_2.5_ level by 10 μg/m^3^ could elevate the risk of AD among residents by 2%-5% (HR = 1.02 to 1.05). Subgroups of male, with older age, with lower the educational level, those who worked as blue-collar workers or farmers before retirement, with a higher BMI (overweight, obesity) were associated with a higher effect of PM_2.5_.

There was a limited but increasing epidemiological evidence regarding the association between particulate air pollution exposure and cognitive functions. A recent study taken by Ma et al. enrolled 31,573 CLHLS participants and 1,131 CABLE participants for cognitive function score using the MMSE. The risk of cognitive decline increased by 10% for every 20 μg/m^3^ increase in PM_2.5_ exposure (HR = 1.10, 95% CI: 1.03–1.18). This study also found that the cognitive decline caused by long-term exposure to PM_2.5_ might be mediated by abnormal amyloid in cerebrospinal fluid, such as Aβ42/Aβ40, P-Tau /Aβ42, Tau /Aβ42, with an intermediate proportion between 17 and 22% [26]. Yuchi et al. found that road proximity was associated with incidence of Alzheimer’s disease, and this association may be partially mediated by air pollution [27]. Michael et al. also reported that higher gestational exposure to PM_2.5_ exposure in the first 16 wk was associated with smaller fetal growth measures, where associations were particularly strong for biparietal diameter (BPD), abdominal circumference (AC), and birth weight [28].

Some studies deliberated findings detailing the mechanisms for a better understanding the relationship between AD and environmental risk factors along with their mechanisms of action on the brain functions. Ultrafine as well as fine PM were proficient to cross into bloodstream and taken up through cells causing mitochondrial damage in addition to oxidative stress, which might be capable to enter the brain directly via the olfactory nerve responsible to AD [29]. Meanwhile, short-term exposure to high intensities of ultra-fine PM was relevant to develop AD, oxidative damage glial cells might increase risk of AD pathogenesis. It was revealed that PM exposure provoked modifications in inflammatory reactions, dendritic spine density loss, decreased hippocampus (CA1 region) dendrite length, increased BACE and Aβ expression, and more amyloid precursor protein (APP) in mice brains to stimulate AD [30]. Therefore, a relation concerning with neuroinflammation as well as exposure of particulate air pollution created a possible pathway in AD risk.

The strengths of the study included that it was one of very few prospective cohort studies on PM_2.5_ exposure influencing AD in eastern China. Some limitations needed to be mentioned in this study. First of all, the PM_2.5_ exposure data used in this study came from high-precision satellite remote sensing models and failed to reflect individual indoor exposure which might lead some exposure measurement errors. Secondly, an important limitation of this work was the use of relatively short-term exposure to assess exposure periods of interest that were potentially much longer, which might impact the analysis. Meanwhile, the yearly exposures showed a decreasing trend, which were similar with other places in China, which might fail to observe the long-term effects of PM_2.5_ exposure. Furthermore, the low participation rate in the target areas might lead a a selection bias, such as the unusual U-shaped distribution of BMI. All of these called for further studies with long-term exposure air pollution and involving longer cohorts to verify our study.

## Conclusion

PM_2.5_ was an important factor that contributed to AD. The suggestion was that to make reducing PM_2.5_ exposure as a means to prevent dementia, especially for the population with older age, with low education levels, and with the profession of farmers or blue-collar workers, and who were overweight and obese. We proposed that the government implemented effective measures to reduce people's exposure to air pollutants such as PM_2.5_, so as to better ensure people's health and prevent the occurrence of AD.

## Supplementary Information

**Additional file 1:** **Supplementary Table 1. **Baseline Information of participants and non-participants.

## Data Availability

The datasets used and analyzed during the current study are available from the corresponding author on reasonable request.
