# Supplementary material for: Associations between PM2.5 exposure and Alzheimer’s Disease prevalence Among elderly in eastern China
Source: Environ Health. 2022 Nov 29;21:119. doi: 10.1186/s12940-022-00937-w (PMC9706836; doi:10.1186/s12940-022-00937-w)
Supplement: Supplementary file 1 — Additional file 1: Supplementary Table 1. Baseline Information of participants and non-participants. [file 12940_2022_937_MOESM1_ESM.doc]

Supplementary Table 1. Baseline Information of participants and non-participants

| **Variable** | **Classification** | **Participants (n=1545)** | **Non-participants (n=197)** | ***P* values** |
| --- | --- | --- | --- | --- |
| Age |  | 68.21 (4.81) | 68.07 (5.30) | 0.52 |
| Age group | 60-64 | 403 (26.08) | 52 (26.40) | 0.99 |
|  | 65-69 | 528 (34.17) | 66 (33.50) |  |
|  | 70-74 | 436 (28.22) | 56 (28.43) |  |
|  | 75-85 | 178 (11.52) | 23 (11.68) |  |
| Marital status | Living alone | 205 (13.65) | 24 (12.18) | 0.57 |
|  | Cohabitation | 1297 (86.35) | 173 (87.82) |  |
| Annual household income | <50,000 | 309 (20.59) | 44 (22.34) | 0.47 |
|  | 50,000-100,000 | 882 (58.76) | 111 (56.35) |  |
|  | ＞100,000 | 310 (20.65) | 42 (21.32) |  |
| Educational degree | Illiteracy | 232 (15.02) | 32 (16.24) | 0.14 |
|  | Primary school | 910 (58.90) | 116 (58.89) |  |
|  | Junior high school | 350 (22.65) | 45 (22.84) |  |
|  | Senior high school and above | 53 (3.40) | 4 (2.03) |  |
| Occupation before retirement | Farmers | 739 (47.83) | 120 (60.91) | 0.002 |
|  | Blue-collar workers | 705 (45.63) | 65 (32.99) |  |
|  | White-collar workers | 101 (6.54) | 12 (6.09) |  |
| BMI, kg/m^2^ |  | 24.51 (3.02) | 24.06 (4.57) | 0.62 |
| BMI group |  |  |  | 0.43 |
| BMI < 24 | Normal | 689 (45.93) | 93 (47.21) |  |
| BMI ≥24 and < 28 | Overweight | 167 (11.13) | 27 (13.71) |  |
| BMI ≥28 | Obese | 644 (42.93) | 77 (39.09) |  |
| Smokers | No | 1189 (76.96) | 160 (81.22) | 0.18 |
|  | Yes | 356 (23.04) | 37 (18.78) |  |
| ETS | No | 995 (64.40) | 135 (68.53) | 0.25 |
|  | Yes | 550 (35.60) | 62 (31.47) |  |
